# Supplementary material for: Effect of Clinical Parameters on Risk of Death from Cancer after Radical Prostatectomy in Men with Localized and Locally Advanced Prostate Cancer
Source: Cancers (Basel). 2022 Apr 18;14(8):2032. doi: 10.3390/cancers14082032 (PMC9032251; doi:10.3390/cancers14082032)
Supplement: Supplementary file 1 [file cancers-14-02032-s001.zip › cancers-1661798-supplementary.pdf]

cancers MDPI

Supplementary Materials: Effect of clinical parameters on risk of death from cancer after radical prostatectomy in men with localized and locally advanced prostate cancer

Daimantas Milonas, Tomas Ruzgas, Zilvinas Venclovas, Daniele Jonusaite, Aivaras Jonas Matijosaitis, Darius Trumbeckas, Edmundas Varpiotas, Stasys Auskalnis, Darijus Skaudickas, Ramunas Mickevicius, Kestutis Vaiciunas, Jonas Mickevicius and Mindaugas Jievaltas

Table S1. Pathological patient characteristics according age <65 vs. ≥65 years.

Parameter	<65 Years (*n* = 1309)	≥65 Years (*n* = 1112)	*p* Value
Pathological stage (pT), *n* (%)			
T2	875 (66.8)	698 (62.8)	
T3a	345 (26.4)	318 (28.6)	0.080
T3b-4	89 (6.8)	96 (8.6)	
PSA, ng/ml – median (IQR)			
	6.05 (4.5-9)	6.6 (5-10)	<0.001
Lymph node status, *n* (%)			
pN0	331 (25.3)	382 (34.4)	
pN1	37 (2.8)	46 (4.1)	<0.001
Unknown	941 (71.9)	684 (61.5)	
GG after RP, *n* (%)			
1	337 (25.8)	293 (26.5)	
2	727 (55.6)	559 (50.5)	
3	149 (11.4%)	102 (9.2)	<0.001
4	42 (3.2%)	67 (6.1)	
5	53 (4.1%)	85 (7.7)	
Unknown	1 (0.1%)	6 (0.4%)	
Surgical margins status, *n* (%)			
Negative	885 (67.6)	732 (65.8)	
Positive	366 (28.0)	332 (29.9)	0.582
Unknown	58 (4.4)	48 (4.3)	

PSA – prostate specific antigen, GG – International Society of Urological Pathology Grade Groups, RP radical prostatectomy, pN0 – negative lymph node, pN1 – positive lymph node.

Table S2. Cumulative 5- and 10-year mortality from cancer and other causes in all cohort and subgroups stratified according to grade group, age and pathological stage.

	**5-yr mortality (95%CI)**		**10-yr mortality (95%CI)**	
	**Prostate cancer**	**Other causes**	**Prostate cancer**	**Other causes**
GG1				
<65yr + pT2-3a	0.1 (0.03-0.3)	3.8 (3.0-5.3)	0.4 (0.1-1.3)	11.1 (8.4-13.3)
≥65yr + pT2-3a	0.2 (0.06-0.6)	8.1 (6.5-10.9)	1.0 (0.3-2.9)	22.5 (17.7-26.1)
<65yr + pT3b-4	0.4 (0.1-1.5)	8.8 (3.9-13.8)	2.0 (0.6-6.4)	23.8 (11.8-30.1)
≥65yr + pT3b-4	0.9 (0.3-3.6)	18.0 (9.4-24.7)	4.8 (1.4-16.9)	43.2 (25.7-51.5)
GG2				
<65yr + pT2-3a	0.2 (0.1-0.4)	3.6 (2.9-4.8)	1.1 (0.6-1.9)	10.1 (7.9-12.3)
≥65yr + pT2-3a	0.5 (0.3-0.9)	7.5 (6.3-9.9)	2.5 (1.4-4.4)	20.4 (16.5-24.4)
<65yr + pT3b-4	1.0 (0.5-2.3)	7.9 (4.3-10.9)	5.1 (2.7-9.6)	20.1 (11.8-26.2)
≥65yr + pT3b-4	2.5 (1.1-5.3)	16.1 (9.2-21.9)	11.8 (6.3-22.0)	35.2 (25.6-45.7)
GG3				
<65yr + pT2-3a	0.5 (0.2-1.4)	3.1 (1.8-4.9)	2.5 (1.0-6.2)	8.2 (5.1-12.5)
≥65yr + pT2-3a	1.2 (0.5-2.8)	6.5 (4.2-9.8)	5.9 (2.7-12.9)	15.9 (10.7-25.5)
<65yr + pT3b-4	2.5 (0.9-6.6)	6.1 (2.8-10.8)	11.8 (4.1-34.3)	12.4 (7.9-26.2)
≥65yr + pT3b-4	5.8 (2.5-13.5)	11.9 (6.6-20.2)	26.1 (12.3-55.2)	19.3 (17.2-47.5)
GG4				
<65yr + pT2-3a	0.9 (0.4-2.2)	4.8 (2.5-7.8)	4.5 (2.1-10.0)	12.2 (7.7-17.8)
≥65yr + pT2-3a	2.2 (0.9-5.3)	9.9 (6.2-14.2)	10.6 (4.8-23.3)	22.4 (15.1-36.9)
<65yr + pT3b-4	4.4 (1.7-11.9)	9.0 (4.7-14.1)	20.5 (11.1-37.8)	15.7 (12.1-35.3)
≥65yr + pT3b-4	10.3 (4.1-26.2)	16.8 (9.5-29.5)	42.4 (25.9-69.2)	20.1 (16.8-40.2)
GG5				
<65yr + pT2-3a	3.1 (1.4-6.8)	5.8 (2.2-7.5)	14.8 (7.4-29.8)	10.5 (5.9-19.4)
≥65yr + pT2-3a	7.3 (4.3-12.7)	11.3 (4.9-14.8)	32.0 (17.4-58.8)	15.2 (12.8-36.6)
<65yr + pT3b-4	14.4 (7.7-27.1)	6.1 (3.9-14.1)	54.6 (34.3-86.9)	0 (0-36.4)
≥65yr + pT3b-4	31.3 (19.3-50.6)	8.3 (4.4-13.7)	84.9 (69.8-100)	0 (0-59.9)

CI – confidence interval, GG – International Society of Urological Pathology Grade Groups, pT – pathological stage.
